# Circulating γδ T cells in young/adult and old patients with cutaneous primary melanoma

**DOI:** 10.1186/1742-4933-2-2

**Published:** 2005-02-01

**Authors:** Francesca Re, Alessia Donnini, Beatrice Bartozzi, Giovanni Bernardini, Mauro Provinciali

**Affiliations:** 1 Laboratory of Tumor Immunology, Immunology Center, I.N.R.C.A. Res. Dept., Ancona, Italy

**Keywords:** γδT cells, aging, melanoma, human, tumor immunity

## Abstract

**Background:**

In a previous study we demonstrated the existence of numerical and functional alterations of γδ T cells in healthy elderly. Recently, we analysed the involvement of γδ T lymphocytes in malignant melanoma, describing a lower frequency of circulating γδ T cells, an altered pattern of cytokine production, and an impaired in vitro expansion of these cells in primary cutaneous melanoma patients.

**Methods:**

In this study we investigated the existence of numerical and functional alterations of circulating γδ T cells in young/adult and old melanoma patients, comparing the data obtained with age-matched healthy subjects.

**Results:**

We demonstrated that the number of circulating γδ^+ ^T cells was significantly and similarly reduced in young/adult and old melanoma patients and in old healthy subjects in comparison with young healthy donors. The decrease was due to a reduction of Vδ2 T cells whereas the number of Vδ1 T cells was not affected. A higher percentage of γδ^+ ^T cells producing TNF-α was found in old healthy donors, whereas a reduced number of TNF-α-producing γδ^+ ^T cells was present in melanoma patients independently by age. No significant difference was observed in IFN-γ production. After a 10-day in vitro culture, both the percentage and the expansion index of γδ T cells, and in particular of Vδ2 subset, were significantly and similarly reduced both in young/adult and old melanoma patients, and in healthy aged people, in comparison with young/adult healthy subjects.

**Conclusions:**

Our study demonstrates that the numerical and functional impairment of γδ T cells found in melanoma patients is not correlated with age and that it has characteristics very similar to the alterations of γδ T cells found in old healthy subjects. We suggest that a similar impairment of γδ T cell population may be related to the increased susceptibility to tumors present in the elderly as well as in the pathogenesis of malignant melanoma.

## Introduction

T lymphocytes bearing the γδ T cell receptor (TCR) represent a minor population of human peripheral lymphocytes (1–10%), the majority of them expressing the CD3^+^CD4^-^CD8^- ^phenotype [1-4]. The ability of γδ T cells to respond to nonprocessed and nonpeptidic phosphoantigens in a major histocompatibility complex (MHC)-unrestricted manner is an important feature distinguishing them from αβ T cells [5-9]. In human peripheral blood two main populations of γδ T cells have been identified based on the TCR composition. The predominant subset expresses the Vδ2 chain associated with Vγ9 and represent 70% of the circulating γδ T cells in adults, while a minor subset (approximately 30%) expresses a Vδ1-Jδ1 chain linked to a chain different from Vγ9. At birth the Vδ1 population predominates, while in adults there is a shift towards Vδ2 T lymphocytes, probably due to a selective response to environmental stimuli such as commonly encountered bacteria. [10].

Although little is known about the physiologic significance of γδ T cells, their marked reactivity toward mycobacterial and parasitic antigens as well as tumor cells suggests that γδ T cells play a role in the anti-infectious and anti-tumoral immune surveillance [11,4]. Few data are available about the number and the function of γδ T cells and of Vδ1 and Vδ2 subsets in aging. The complexity of the gamma delta T cell repertoire has been found to decrease with age as a consequence of the expansion of a few T cell clones [12]. In our previous paper [11], we have evaluated the role of γδ T cells from young, old, and centenarian subjects, demonstrating an age-dependent alteration of γδ T lymphocytes, with a lower frequency of circulating γδ T cells, an altered pattern of cytokine production, and an impaired in vitro expansion of these cells. We suggested a role of the γδ T cell impairment in the age-related increase of infections and tumor diseases. Other studies have showed the involvement of γδ T cells in the immune defence against cancer either through a direct reactivity against tumor cells, or because of their regulatory interactions with αβ T cells [13]. Recently, we described an impairment of γδ T cell population in patients with cutaneous primary melanomas, with a decrease of their absolute number and percentage, an altered cytokine production, and a reduced expansion of γδ T cells, and particularly of the Vδ2 subset [14].

On the basis of the pivotal role that γδ T cells may have in the elderly and in the immune response against melanoma we tried to find out a possible correlation in the alteration of γδ T cells between aged people and melanoma patients. In this study we evaluated the peripheral representation, the in vitro expansion, and cytokine production γδ T cells from young/adult and old melanoma patients, comparing the results with those obtained in age-matched healthy controls.

## Materials and Methods

### Cell preparation and stimulation

Human peripheral blood was obtained from 9 young (mean age ± SD, 42.3 ± 9.4 years; median: 41.0 years, range 30–59), 12 old (71.8 ± 5.4 years; median 72.5 years, range 60–80) melanoma patients, and 10 young (39.0 ± 5.7 years; median: 38.5, range 30–55), and 13 old (74.0 ± 2.0 years; median: 74.0 years, range 60–80) healthy donors. Healthy subjects were volunteers in good and stable clinical conditions, and had laboratory parameters in the physiological range. We excluded subjects in poor health with degenerative diseases or in therapy with drugs interfering with the immune system. Melanoma patients have been admitted to the Dermatology Unit of the I.N.R.C.A. Hospital of Ancona. Melanoma patients were in good health other than for the existence of melanoma as checked on the basis of clinical and laboratory parameters. The investigations were performed after approval by a local institutional review board. A written informed consent was obtained from each subject. Diagnosis of melanoma was histologically confirmed. All patients brought cutaneous primary non-metastatic melanoma and were staged according to the new American Joint Committee on Cancer staging system for cutaneous melanoma [15]. A blood drawing was taken before the surgical excision. Each donor was tested once and all the tests were carried out with a single blood sample.

Fresh peripheral blood mononuclear cells (PBMC) were fractionated on Ficoll-Paque (Pharmacia, Uppsala, Sweden) and separated by density gradient centrifugation (400 g, 30 min). Cells from the interface of the gradients were washed twice with Ca^2+ ^and Mg^2+^- free phosphate buffered saline (PBS, Gibco/Life Technologies, Gaithersburg, MD, USA) and resuspended in RPMI 1640 supplemented with 10% heat-inactivated fetal bovine serum, penicillin (100 U/ml) and streptomycin (100 μg/ml) (all from Life Technologies, complete medium) at a concentration of 1.5 × 10^6^/ml. Mononuclear cells were cultured in the complete medium supplemented with 100 U/ml of IL-2 (Chiron Italia, Milan, Italy). Phosphoantigen-specific stimulation of γδ T cells was performed using the nonpeptidic antigen isopentenylpyrophosphate (30 μg/ml, IPP, Sigma Chemical Co., St. Louis, MO, USA). The cells were incubated at 37°C in an atmosphere of 95% air, 5% carbon dioxide, at 90% relative humidity in 24 well plates.

### Monoclonal antibodies and FACS analysis

PBMCs were analysed for cell phenotype through double staining with the following monoclonal antibodies (mAbs): anti-CD3 (PE) and anti-pan γδ T cells (FITC), or anti-TCR Vδ1 or anti-TCR Vδ2. The phycoerythrin (PE) -conjugated monoclonal antibody anti-CD3 was purchased from EuroClone (Devon, UK). The fluorescein isothiocyanate (FITC) -conjugated anti-pan TCR γδ, anti-TCR Vδ1, and anti-TCR Vδ2 were purchased from Endogen (Boston, MA, USA). IgG1 (Becton Dickinson) was used as isotype control.

0.5 × 10^6 ^PBMCs were washed in PBS containing 0,1% NaN_3 _plus 5% FBS and labelled with 5 μl of anti-CD3 or anti-TCR Vδ1 MoAbs or 2.5 μl of anti-pan TCR γδ or anti-TCR Vδ2 for 30 min in ice. At the end of the incubation, cells were washed in PBS containing 0,1% NaN_3_, resuspended in PBS (Gibco) and immediately analysed with a Coulter XL flow cytometer.

### Intracellular detection of IFN-γ and TNF-α

Mononuclear cells were stimulated with IPP and IL-2 for 18 h, and GolgiPlug (a protein transport inhibitor containing brefeldin A, PharMingen, Milton Keynos, England) was added during the last 12 h of culture to block intracellular transport processes and allow cytokine accumulation. 0.5 × 10^6 ^stimulated cells were stained with the anti-pan TCR γδ mAb for 30 min at 4°C. Fixation-permeabilization of cells was performed in PBS/2% paraformaldehyde for 15 min at 4°C, followed by incubation for 30 min at room temperature in the dark with PE-conjugated anti-human IFNγ mAb or anti-human TNF-α mAb diluted in PBS, 1% BSA, and 0.05% saponin. Cells were finally washed twice in PBS, 1% BSA, and 0.01 % saponin and analysed on a XL flow cytometer (Coulter).

### Expansion assay

PBMC were cultured for up to 10 days in the complete medium supplemented with 100 U/ml of IL-2 and 30 μg/ml of IPP to determine a phosphoantigen-specific stimulation of γδ T cells. After 1 wk of culture, the volume corresponding to half the culture medium was replaced by fresh medium. On day 10 of culture viable cells were determined by trypan blue exclusion and used for FACS analysis. The viability was always greater than 98% as determined by trypan blue exclusion. The expansion of γδ T cells was followed by cytometric analysis through double staining of stimulated cells with anti-CD3 (PE) and anti-pan γδ, or anti Vδ1, or anti Vδ2 T (FITC) mAbs. The absolute number of γδ T cells in each culture was calculated as follow: (percentage of γδ T cells among total cells) × (total cell count)/100. The γδ T cell expansion index was then calculated by dividing the absolute number of γδ T cells in stimulated cultures by the absolute number of γδ T cells before culture [16].

### Statistical analysis

Data were analysed for statistical significance by using parametric or nonparametric tests according to the distribution of the data. Comparisons of variables among groups were made by one-way analysis of variance (ANOVA) or Kruskal-Wallis one-way ANOVA on ranks. When significant differences were found, the differences among groups were made by the Student-Newman-Keuls method or Dunn's method. Difference between means was considered significant at the 5% level (*P *< 0.05). The statistical analysis was performed with SigmaStat software version 1.03 (Jandel Scientific, Germany).

## Results

### Ex vivo analysis of γδ T lymphocytes

**Figure 1:**
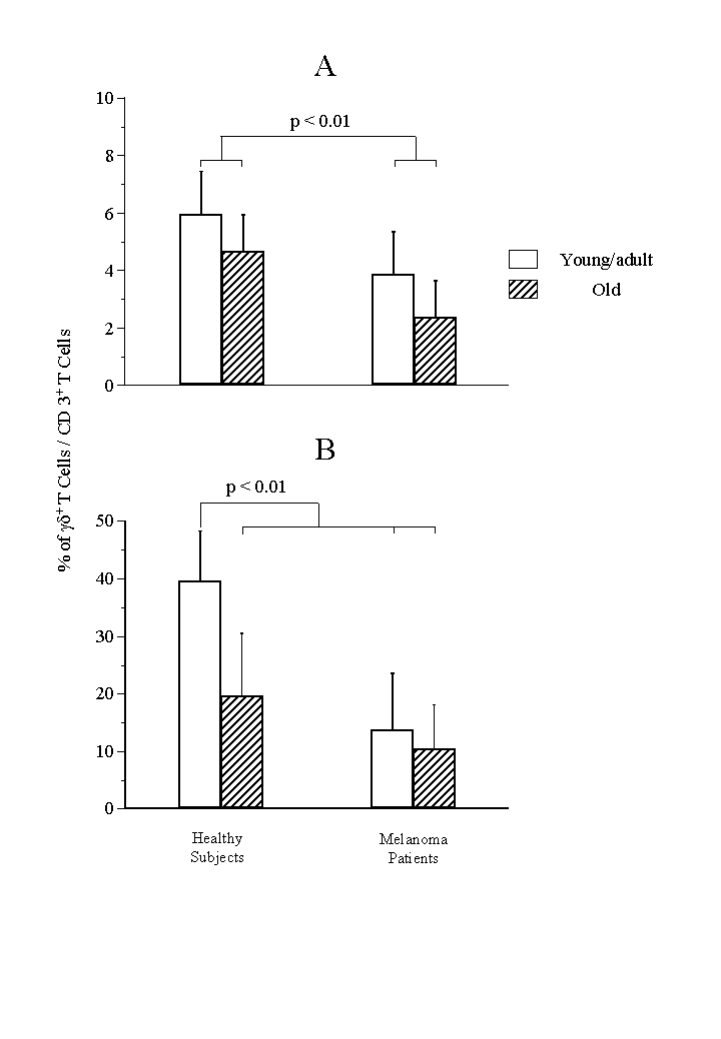
Percentage of γδ T cells in young/adult and old melanoma patients and age-matched healthy subjects. Freshly isolated (A) or 10-day cultured (B) PBMC from young/adult and old melanoma patients and young/adult and old healthy subjects were double stained with MoAb anti pan- γδ (FITC) and anti-CD3 (PE) and analyzed by flow cytometry. Statistical analyses was performed as reported in Mat. and Methods.

Peripheral blood lymphocytes from 9 young/adult and 12 old melanoma patients and 10 young and 13 old healthy subjects were analysed for the percentage and the absolute number of γδ T cells through double staining with anti-CD3 and anti-γδ mAbs. As shown in Table 1 the absolute number of γδ T cells was significantly reduced in both groups of melanoma patients and in healthy aged people in comparison with young/adult healthy subjects (p < .01). As shown in Fig. 1A, the ex vivo percentage of CD3^+^γδ^+ ^T cells in the peripheral blood was significantly lower in melanoma patients than in healthy donors (p < .01). As shown in Table 1, the absolute number of Vδ1 T cells did not show significant difference in the four groups of donors. Differently, the absolute number of Vδ2 T cells was significantly reduced in both groups of melanoma patients and in old healthy people in comparison with young/adult healthy subjects (p < .0001). The Vδ2 and Vδ1 subsets were differently represented in the four groups: in young/adult healthy controls the Vδ2 subset was predominant (Vδ2/Vδ1 ratio = 2.2) whereas in old healthy donors and in young/adult and old melanoma patients the Vδ2/Vδ1 ratio was progressively decreased.

**Table 1:** Absolute number of γδ T cells, Vδ1 T cells, and Vδ2 T cells, in young/adult and old healthy subjects and melanoma patients.

	**Absolute number**			
	
*Donors*	**γδ T cells**	**Vδ1 T cells**	**Vδ2 T cells**	**Vδ2/Vδ1 ratio**
				
	
Healthy				
	
*Young/adult*	115.2 ± 39.3^a^	38.0 ± 11.9	82.6 ± 34.0	2.2
				
*Old*	62.1 ± 28.7*	35.9 ± 11.7	37.7 ± 24.6*	1.0
				
	
Melanoma				
	
*Young/adult*	74.5 ± 25.3*	31.9 ± 18.1	42.8 ± 9.8*	1.3
				
*Old*	52.9 ± 38.0*	20.6 ± 3.7	33.5 ± 31.2*	1.6

^a ^Data are expressed as mean ± S.D. of the number of cells per mm^3 ^in the peripheral blood.

*p at least <0.01 versus young/adult healthy subjects

### Cytokine production by γδ T lymphocytes

**Figure 2:**
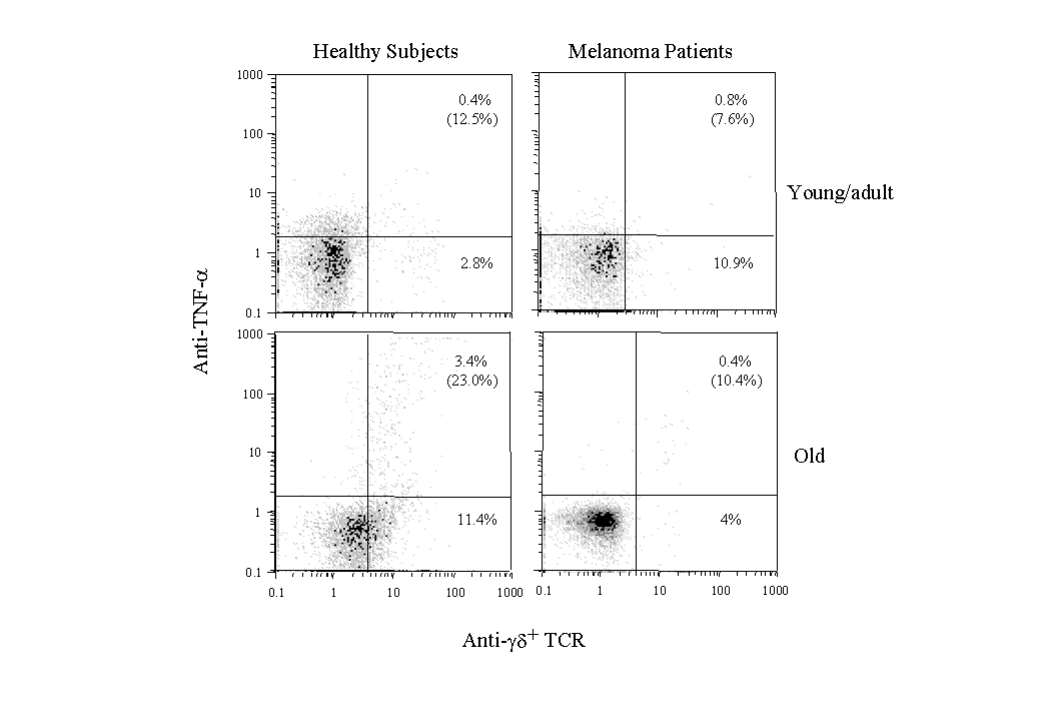
Analysis of TNF-α production by γδ T cells in melanoma patients and age-matched healthy subjects. PBMC young/adult and old melanoma patients and young/adult and old healthy subjects were stimulated for 18 h in the presence of IPP (30 μg per ml) and IL-2 (100 U per ml). The last 12 h of culture were performed in the presence of GolgiPlug, a protein transport inhibitor containing brefeldin. Single-cell analysis of TNF-α synthesis in γδ T cells from a representative subject for each group was performed following dual staining with cell surface anti- γδ T (FITC) MoAb and intracellular anti- TNF-α (PE) MoAb. Number in brackets indicate the percentages of γδ T cells synthesizing TNF-α among total γδ T lymphocytes.

Since it has been demonstrated that activated γδ T cells produce TNF-α and IFN-γ, we studied the intracellular production of these cytokines in one-day stimulated γδ T cells from healthy subjects and melanoma patients. As shown in Fig. 2 the percentage of γδ T cells producing TNF-α was significantly higher in old healthy controls in comparison with young/adult healthy, and young/adult and old melanoma patients (p < .05). The percentage of γδ T cells producing IFN-γ was similar in young/adult and old healthy subjects (mean ± SD, 17.9 ± 10.0 and 14.7 ± 8.9). In a similar way the percentage of γδ T cells producing IFN-γ did not show differences between young/adult and old melanoma subjects (8.5 ± 4.9 and 8.1 ± 1.3) (data not shown).

### Expansion of γδ T lymphocytes

The expansion of γδ T cells was evaluated after 10 days of culture in the presence of IPP and low dose interleukin-2 (IL-2). Both the proportion of γδ T cells, evidenced by double staining FACS analysis, and their relative increase in comparison with the γδ T cell number found on day 0 (expansion index), were evaluated. As shown in Fig. 1B, the proportion of γδ T cells reached on day 10 was significantly lower in both groups of melanoma patients and in old healthy subjects than in young/adult healthy donors (p < .01). In a similar way, the expansion index of γδ T cells after 10 days of in vitro culture was significantly reduced in the same three groups mentioned above (p < .05, Table 2). As shown in the same Table 2, the expansion index of the Vδ2 subset was significantly lower in all melanoma patients and in old healthy donors than in young healthy donors (p < .03).

**Table 2:** Expansion index of γδ T cells and Vδ2 T cells in healthy subjects and melanoma patients

	**Expansion Index^a^**	
	
**Donors**	**γδ T cells**	**Vδ2 T cells**
		
Healthy		

*Young/adult*	13.1 ± 8.8^b^	7.1 ± 4.5
*Old*	4.6 ± 3.5*	3.1 ± 2.0*
		

Melanoma		

*Young/adult*	3.8 ± 2.6*	4.6 ± 0.2*
*Old*	2.0 ± 1.8*	0.9 ± 0.4*

^a ^The expansion index was calculated by dividing the absolute number of γδ T cells in stimulated cultures by the absolute number of γδ T cells before culture.

^b^Data are expressed as mean ± S.D.

*p at least <0.05 versus young/adult healthy subjects

## Discussion

We and others have demonstrated an impaired potential of γδ T cells in aged people, as evidenced by the reduction of the absolute number of circulating γδ T cells, in particular of the Vδ2 T subset, an altered pattern of cytokine production, an impaired in vitro expansion of these cells, and an increased expression of the early activation marker CD69, in aged people in comparison with young subjects [11,17,18]. Recently, studying a group of melanoma patients ranging from young to old age (32–80 yr), we described numerical and functional alterations of γδ T cells from these subjects, once compared to healthy age-matched donors [14]. In this study we have investigated on whether the age-related impairment of circulating γδ T cells is similar to the alteration found in melanoma patients and if melanoma patients of advanced age have a greater impairment of γδ T cells in comparison with melanoma patients of younger age or with old healthy donors. With these premises, we studied the peripheral representation, in vitro expansion, and cytokine production of γδ T lymphocytes from young/adult and old patients with cutaneous primary melanoma comparing the data obtained with age-matched healthy subjects.

We demonstrated that both the number of circulating γδ T cells and their in vitro expansion were decreased in melanoma patients and that the impairment did not correlate with the age of patients. Young/adult and old melanoma patients had a similar derangement of γδ T cells, and this impairment had numerical and functional characteristics like to those found in old healthy subjects. This evidence stresses the relevant role that this lymphocyte population may exert, either directly or through the regulation of T cell-mediated specific responses [11], both in the elderly and in melanoma patients.

The reduction of γδ T cell number well correlated with the decrease of the Vδ2 T cell subset, i.e., the most frequent subset of circulating γδ T cells [2,4]. The Vδ2 population is involved in the reactivity toward microbial antigens and tumor cell antigens [4,19]. The role of Vδ2 T cells in the immune defence against cancer has been demonstrated on the basis of their reactivity against certain lymphoma cells, such as Daudi cells [20], and for their presence among tumor infiltrating lymphocytes in various cancer types [21]. Not only the number but also the function of γδ T cells was altered in melanoma patients as well as in old healthy subjects. The in vitro expansion of γδ T cells, that represent one of the most relevant functional parameters for γδ T cells, was significantly reduced in young/adult and old melanoma patients, and in old healthy donors.

Under normal conditions, γδ T cells respond to antigen challenge by secreting large quantities of TNF-α and IFN-γ [16,21] which contribute to the activation of both specific and aspecific immune responses. In aged subjects we found an increased production of TNF-α by γδ T cells [11]. In this study, we show that the percentage of γδ T cells producing TNF-α was significantly reduced in young/adult and old melanoma patients in comparison with age-matched healthy subjects. Probably, the pro-inflammatory state which has been described in old ages [22], may represent a stimulus for the production of TNF-α in γδ T cells from aged subjects, differently with what occurs in old melanoma patients.

In conclusion, we demonstrate that the numerical and functional derangement of γδ T cells which we have found in melanoma patients, is not correlated with age of donors, and that old patients with cutaneous primary melanoma have an impairment of γδ T cells similar to that found in old healthy subjects. This evidence suggests a link between γδ T cell deterioration and the low protection against infections and tumor diseases present in the elderly, as well as the inefficacious immune defense against melanoma, both in young/adult and old ages.
